# Multiple Group Decision Making for Selecting Emergency Alternatives: A Novel Method Based on the LDWPA Operator and LD-MABAC

**DOI:** 10.3390/ijerph17082945

**Published:** 2020-04-24

**Authors:** Xia Liang, Fei Teng, Yan Sun

**Affiliations:** School of Management Science and Engineering, Shandong University of Finance and Economics, Jinan 250014, China; liangxia@sdufe.edu.cn (X.L.); tf1049158564@163.com (F.T.)

**Keywords:** emergency decision, multiple groups, linguistic distribution, prior criteria

## Abstract

When an emergency event occurs, it is critical to respond in the shortest possible time. Therefore, the rationality and effectiveness of emergency decisions are the key links in emergency management. In this paper, with aims to investigate the problem of emergency alternatives selection, in which a large number of experts from multiple groups consider the linguistic evaluations of emergency alternatives and prior orders of criteria, a novel emergency decision method is proposed. First, many experts from multiple subgroups are employed to evaluate the emergency alternatives associated with multiple criteria in the format of linguistic terms. Then, linguistic distribution evaluations for the emergency alternatives of the criteria associated with each subgroup are constructed. With respect to the linguistic distribution evaluations associated with the different subgroups, the linguistic distribution power average (LDPA) and linguistic distribution weighted power average (LDWPA) operators are developed so as to aggregate the subgroups’ evaluations. Next, based on the linguistic distribution multi-attributive border approximation area comparison (LD-MABAC) method, the distance matrix of the emergency alternatives is calculated. Furthermore, the prior weights of the criteria are determined based on the distance values. Finally, the ranking result of the emergency alternatives is derived. A practical example of emergency alternatives selection is adopted to illustrate the availability and practicability of the proposed method.

## 1. Introduction

In recent years, various emergency events have frequently occurred, such as the Wenchuan earthquake in China and the 9/11 terrorist attack in the USA. Such emergency events have disrupted social stability and economic development, often with unexpected catastrophic consequences. As kinds of low-probability and high-risk events, emergencies often have the characteristics of uncertainty and irregularly, and are highly destructive, seriously threatening or affecting the security of people’s lives and property. When an unexpected emergency event occurs, emergency decision makers must respond and implement effective emergency actions in the shortest possible time to reduce and mitigate the casualties and property losses [1,2,3,4]. Due to the increasing number of emergencies in recent years, the requirements for the emergency response ability of emergency management groups have gradually improved. How to effectively carry out emergency strategies, reduce the harm of emergencies to human society as much as possible, and ensure the safety of life and property is a very challenging task [5,6,7]. In emergency management, how to choose and implement the most reasonable emergency plan is an important area of research.

However, it is an intractable task to determine the emergency action in practical emergency responses. On the one hand, due to the complexity of emergency response tasks, many experts from one or multiple groups are often required to participate. Since there are differences in the benefits of group experts, the preferences of emergency alternatives associated with experts are different. Therefore, it is necessary to research emergency decision problems that involve a great many experts. On the other hand, due to the time urgency of emergency decision making, the characteristics of the qualitative criteria, and the limitations of the expert’s knowledge and experience, many experts from multiple groups usually think that it is more reasonable to use imprecise information, such as linguistic information, to express evaluations of emergency alternatives [8,9]. In addition, the weights of the criteria are unknown, which only involve prior orders. Hence, emergency decision problems widely exist in practical decision problems, and have attracted the attention of many scholars. At present, many domestic and foreign scholars have studied the problem of emergency alternatives selection and obtained some research results.

Therefore, some effective results have been obtained in research on emergency decisions with a home or abroad influence. Prior research has resulted in abundant achievements for emergency decision analysis. Psychological research has shown the importance of the emergency decision maker’s behavior in the decision process, which indicates that emergency decision makers are usually bounded by rationality. In some of the existing research, the behaviors of emergency decision makers were considered, such as the reference dependent, loss aversion, and regret aversion, etc. [10,11,12]. With respect to the risk emergency decision problem, considering the reference dependence and loss aversion behaviors of the decision maker, Liu et al. [10] proposed a method based on prospect theory. Passos et al. [11] investigated the oil spill emergency problem. Then, a TODIM-FSE decision support method, which combines the characteristics of an acronym in Portuguese of interactive and multi-attribute decision making (TODIM) and fuzzy synthetic evaluation (FSE) methods, was proposed to solve the problem of the oil spill emergency response. With respect to the emergency decision problem with interval number evaluations, considering the psychological behavior of emergency decision makers, Li and Cao [12] presented a risk TODIM decision analysis method. The case-based reasoning (CBR) method is an effective method that can handle emergency decision problems. With respect to the emergency decision-making problem, Fan et al. [5] proposed a novel method for the hybrid similarity measure with some formats of hybrid evaluations. Liao et al. [13] developed environmental emergency preparedness systems by using CBR technology and the improved genetic algorithm (IGA). Gao et al. [14] proposed a probability correction method by using the CBR method to obtain more accurate and reasonable probabilistic linguistic evaluations, so as to propose a novel emergency decision method. 

In the emergency decision process, due to the complexity of decision problems, some experts may be employed to participate in decision making. Therefore, emergency group decision making involving many experts has been the focus of attention of many researchers. Yu and Lai [6] proposed a method for determining the degree of importance of experts from the perspective of group consistency, and a distance-based group decision-making method, aiming at the multi-index emergency decision-making problem with irregular multi-person participation. For the multi-index group decision-making problem of emergency plan selection, Ju et al. [2] proposed a group decision-making method of combining Dempster–Shafer evidence theory with the analytic hierarchy process (DS/AHP) and technique for order preference by similarity to an ideal solution (TOPSIS). Then, Ju et al. [7] investigated emergency alternative evaluations and selection problems with 2-tuple linguistic evaluations, and presented a novel emergency decision method of combining the analytic network process (ANP) method, the decision-making trial and evaluation laboratory (DEMATEL) technique, and the 2-tuple linguistic TOPSIS technique. In order to determine the optimal technique scheme, Qu et al. [15] presented a multi-stage technical screening and evaluation tool. Li and Wei [9] investigated an emergency decision problem with multiple decision makers, which was operational, and proposed a novel emergency decision method based on DS evidence theory and the probabilistic linguistic weighted averaging operator. Gao et al. [16] investigated an emergency decision problem with incomplete probabilistic linguistic preference relations. Then, an incomplete probabilistic linguistic term set (InPLTS), and a consistency-based emergency decision method, was proposed. Xu et al. [17,18] investigated the large group emergency decision-making problem with preference information. Then, emergency decision methods based on the clustering algorithm, average consistency, and standard deviation were presented.

From the existing research results, although preliminary results have been obtained in the study of emergency plan selection considering the behavior of the decision makers, there are still some problems worthy of further study. On the one hand, most research on emergency decision making has mainly considered one or fewer experts involved in the decision process. Even fewer studies have considered different groups of participants. On the other hand, most research on emergency decision making has only considered the case in which the criteria weights are known, and little research on criteria with a priority order and unknown weights has been conducted. In fact, in many practical problems of emergency alternatives selection, criteria often have a priority order. Therefore, it is very necessary to conduct a study to determine the criteria priority weight according to the priority order relation of the criteria and the evaluation result of the emergency alternatives, and to further rank and select emergency alternative(s).

Compared with traditional emergency alternatives selection, in which a great many experts from multiple groups participate, the evaluation or selection problem oriented on linguistic evaluations is more complex. Therefore, the goal of this study is to propose a decision support model to rank and select desirable emergency alternative(s). The contributions of this study are as follows:

(1) To conduct linguistic evaluations provided by experts from multiple subgroups. Based on probability theory and statistics theory, linguistic distribution evaluations for emergency alternatives of each criterion associated with each subgroup can be constructed.
(2) With respect to the situation of multiple subgroups, participation provides different linguistic evaluations, based on the linguistic distribution power average (LDPA) and linguistic distribution weighted power average (LDWPA) operators, in comparison to a method for aggregating extreme data associated with different subgroups.
(3) Considering the prior order of the criteria of emergency alternatives with linguistic evaluations, the prior weights of criteria are determined. Most important of all, a reasonable and effective method based on a new linguistic distribution multi-attributive border approximation area comparison (LD-MABAC) method for ranking emergency alternatives is proposed.

The structure of this paper is as follows. In Section 2, some basic concepts of linguistic distribution evaluations and the MABAC method are introduced. In Section 3, we propose a novel operator associated with linguistic distribution information. Then, the problem formulation and resolution process are represented in Section 4. In Section 5, employing many linguistic terms provided by experts from multiple groups, the linguistic distribution matrix for emergency alternatives associated with the subgroups is constructed. Then, the subgroup linguistic distribution matrix is calculated based on the linguistic distribution weighted power operator. By the methods of LD-MABAC and criteria prior orders, the ranking result of the emergency alternatives is subsequently obtained. In Section 6, an example of selecting urban flood prevention alternatives is presented to illustrate the feasibility and effectiveness of the proposed method. Finally, conclusions are given in Section 7.

## 2. Materials and Methods

In this section, some basic knowledge on the linguistic term, distribution linguistic variable, and MABAC method are introduced, which will be used throughout this study.

### 2.1. Linguistic Distribution

Fuzzy evaluations, stochastic evaluations, and linguistic evaluations are useful uncertain evaluations in practical decision making [19,20,21,22]. As a valid form for describing uncertain evaluation, linguistic evaluations can represent qualitative evaluations more realistically. Herrera [23,24] proposed a finite and totally ordered linguistic term set $S=\{s_{0},s_{1},\cdots ,s_{\tau }\}$ with odd cardinality. Additionally, the terms in S are placed symmetrically. For example, a linguistic set S with seven terms could be shown as. $S=\{s_{0}=terrible,\;s_{1}=very\;low,s_{2}=low,\;s_{3}=ordinary,s_{4}=high,s_{5}=very\;high,$ $s_{6}=perfect\}$. Usually, it is required that the linguistic term set S satisfies the following characteristics: (a) There is a negative operator, i.e., $Neg(s_{r})=s_{\tau -r}$, where τ+1 is the cardinality of S, and (b) the linguistic terms are ordered in an ascending manner in S, i.e., $s_{r}\leq s_{r{}^{\prime }}\Leftrightarrow r\leq r{}^{\prime }$. Therefore, there exists a maximization operation, i.e., if $s_{r}\geq s_{r{}^{\prime }}$, then $\max\{s_{r},s_{r{}^{\prime }}\}=s_{r}$, and a minimization operator, i.e., $\min\{s_{r},s_{r{}^{\prime }}\}=s_{r}$ when $s_{r}\leq s_{r{}^{\prime }}$.

In order to preserve all of the given evaluation information, Xu [25,26] extended the discrete term set $S=\{s_{0},s_{1},\cdots ,s_{\tau }\}$ to a continuous linguistic term set $\bar{S}=\{s_{\alpha }|s_{0}\leq s_{\alpha }\leq s_{\tau },\alpha \in [0,\tau ]\}$. Here, if $s_{\alpha }\in S$, then $s_{\alpha }$ is an original linguistic term. If $s_{\alpha }\in \bar{S}$, then $s_{\alpha }$ is a virtual linguistic. Generally, the linguistic terms provided by the decision maker are in the format of original linguistic terms. We suppose that $s_{\alpha },s_{\beta }\in \bar{S}$ are two linguistic terms, and $\mu ,\mu_{1},\mu_{2}\in [0,1]$ are three parameters, so the operational laws of linguistic terms can be shown as follows:

(1) $s_{\alpha }⊕s_{\beta }=s_{\alpha +\beta }=s_{\beta }⊕s_{\alpha }\;$;
(2) $\mu s_{\alpha }=s_{\mu \alpha }$;
(3) $(\mu_{1}+\mu_{2})s_{\alpha }=\mu_{1}s_{\alpha }⊕\mu_{2}s_{\alpha }$;
(4) $\mu (s_{\alpha }⊕s_{\beta })=\mu s_{\alpha }⊕\mu s_{\beta }$.

The linguistic distribution, as a useful generalized format of linguistic term evaluation, is widely applied in multiple criteria decision making [27,28,29,30]. The definition of the linguistic distribution evaluation is introduced in the following.

**Definition** **1** [27,28]**.** *Suppose* $S=\{s_{0},s_{1},\cdots ,s_{\tau }\}$ *is a linguistic term set, where* $s_{r}\in S$*,* r=0,1,⋯,τ*. Let* $p_{r}$ *be the symbolic proportion of linguistic term* $s_{r}$*, which is subject to* $0\leq p_{r}\leq 1$ *and* $\sum_{r=0}^{\tau }p_{r}=1$*. Then, an assessment* $\varphi =\{(s_{r},p_{r})|r=0,1,\cdots ,\tau \}$ *is termed a linguistic distribution evaluation in linguistic term set* S.

**Definition** **2** [27,29]**.** *Let* $\varphi =\{(s_{r},p_{r})|r=0,1,\cdots ,\tau \}$*, where* $s_{r}\in S$*,* $0\leq p_{r}\leq 1$*, which is a linguistic distribution evaluation of* $S=\{s_{0},s_{1},\cdots ,s_{\tau }\}$*. The expectation of* φ is defined as follows:

(1) $$E(\varphi )=\sum_{r=0}^{\tau }p_{r}s_{r}.$$

Then, some operational laws for linguistic distribution evaluation in $S=\{s_{0},s_{1},\cdots ,s_{\tau }\}$ are developed, which are in the following.

(1) A comparison operation: Let $\varphi_{1}$ and $\varphi_{2}$ be two linguistic distribution evaluations in $S=\{s_{0},s_{1},\cdots ,s_{\tau }\}$. Then,
  (a) if $E(\varphi_{1})<E(\varphi_{2})$, $\varphi_{1}$ is smaller than $\varphi_{2}$, denoted as $\varphi_{1}<\varphi_{2}$, and
  (b) if $E(\varphi_{1})=E(\varphi_{2})$, then $\varphi_{1}$ and $\varphi_{2}$ are equal, denoted as $\varphi_{1}∼\varphi_{2}$.
(2) For linguistic distribution evaluation, there is a negation operator that is shown as
  $$Neg\left(\left\{(s_{r},p_{r})|r=0,1,\cdots ,\tau \right\}\right)=\{(s_{\tau -r},p_{r})|r=0,1,\cdots ,\tau \}$$

In order to measure the degree of deviation between two linguistic distribution evaluations, motivated by the distance measure developed by Liang et al. [31], in the following, the definition of the distance between two linguistic distribution evaluations is presented.

**Definition** **3.** *Let*$\varphi_{1}$*and*$\varphi_{2}$*be two distribution linguistic evaluations. Then, the normalized distances of*$\varphi_{1}$*and*$\varphi_{2}$ are defined as

(2) $$d(\varphi_{1},\varphi_{2})=\sqrt{\frac{1}{2(\underset{ij}{\max}y_{ij}-\underset{ij}{\min}y_{ij})}[P(\varphi_{1})-P(\varphi_{2})]Y{\left[P(\varphi_{1})-P(\varphi_{2})\right]}^{T}},$$

*where* $P(\varphi_{k})={\left[p_{0}^{k},p_{1}^{k},\cdots ,p_{\tau }^{k}\right]}^{T}$*;* k=1,2 *; and the matrix* Y *is defined as* $Y={\left[y_{ij}\right]}_{T\times T}$*,* $y_{ij}={\left[\Delta^{-1}(s_{\tau })\right]}^{2}-{\left[\Delta^{-1}(s_{i})-\Delta^{-1}(s_{j})\right]}^{2}$*,* $\Delta^{-1}(s_{i})=i+1$*. Matrix* Y *is a positive symmetric matrix. Obviously, the larger the deviation degree between* $\varphi_{1}$ *and* $\varphi_{2}$ *is, the smaller the value of* $y_{ij}$ is.

With regard to the distance of two linguistic distribution evaluations, some properties are provided in the following.

**Property 1** **(Boundary).** *For any two linguistic distributions*$\varphi_{1}$*and*$\varphi_{2}$*,*$0\leq d(\varphi_{1},\varphi_{2})\leq 1$*and*$d(\varphi_{1},\varphi_{2})=0\Leftrightarrow \varphi_{1}=\varphi_{2}$.

**Property 2** **(Symmetry).** *For any two linguistic distributions*$\varphi_{1}$*and*$\varphi_{2}$*,*$d(\varphi_{1},\varphi_{2})=d(\varphi_{2},\varphi_{1})$.

**Property 3** **(Degeneration).** *If*$\varphi_{1}$*and*$\varphi_{2}$ are two special linguistic distribution evaluations, i.e., two linguistic terms, then the distance between $\varphi_{1}$ *and* $\varphi_{2}$ is degraded into the Euclidean distance of the subscripts.

### 2.2. MABAC Method

Suppose that there is a multiple criteria decision problem. Let M={1,2,⋯,m} and N={1,2,⋯,n} be the index set of alternatives and criteria, respectively. We suppose that set $A=\{A_{1},A_{2},\cdots ,A_{m}\}$ is a finite set of alternatives, and $C=\{C_{1},C_{2},\cdots ,C_{n}\}$ is a finite set of criteria. Let $W={\left(w_{1},w_{2},\cdots ,w_{n}\right)}^{T}$ and $w_{j}\geq 0$ for j∈N be the criteria weight vector, where $w_{j}$ is the weight assigned to criterion $C_{j}$. Without a loss of generality, we suppose that all the criteria are of the benefit type. Let $B={\left[b_{ij}\right]}_{m\times n}$ be the decision matrix, where $b_{ij}$ is the evaluation of alternative $A_{i}$ with respect to criterion $C_{j}$.

The multi-attributive border approximation area comparison (MABAC) is a new decision-making method, which was originally proposed by Pamučar and Ćirović [32] and has been applied by some investigations [33,34]. In the following subsection, complete implementation of the MABAC method is introduced in detail.

**Step 1.** In order to unify dimensions, the decision matrix $B={\left(b_{ij}\right)}_{m\times n}$ should be normalized into the normalized matrix $B{}^{\prime }={\left(b_{ij}^{{}^{\prime }}\right)}_{m\times n}$, where $x_{ij}$ is built by

(3) $$b_{ij}^{{}^{\prime }}=\frac{b_{ij}-\underset{i\in M}{\min}\{b_{ij}\}}{\underset{i\in M}{\max}\{b_{ij}\}-\underset{i\in M}{\min}\{b_{ij}\}},\;i\in M,\;j\in N.$$

**Step 2.** Since the different criteria have different weights, the weighted decision matrix $X={\left(x_{ij}\right)}_{m\times n}$ can be computed by using the formula

(4) $$x_{ij}=w_{j}b_{ij}^{{}^{\prime }},\;i\in M,\;j\in N,$$

where $w_{j}$ is the weight of the criterion $C_{j}$.

**Step 3.** The border approximation area (BAA) vector $H=(h_{1},h_{2},\cdots ,h_{n})$ needs to be calculated, where $h_{j}$ is calculated by

(5) $$h_{j}={\left(\prod_{i=1}^{m}x_{ij}\right)}^{1/m},\;j\in N.$$

**Step 4.** Furthermore, we can construct the distance matrix $D={\left(d_{ij}\right)}_{m\times n}$, where $d_{ij}$ is obtained by

(6) $$d_{ij}=\left\{ \begin{array}{ll} d(x_{ij},h_{j}), & if\;x_{ij}≻h_{j}\\ 0, & if\;x_{ij}=h_{j}\\ -d(x_{ij},h_{j}), & if\;x_{ij}≺h_{j} \end{array}\right.,\;i\in M,\;j\in N,$$

where $d(x_{ij},h_{j})$ expresses the distance between the values $x_{ij}$ and $h_{j}$ in H.

**Figure 1:**
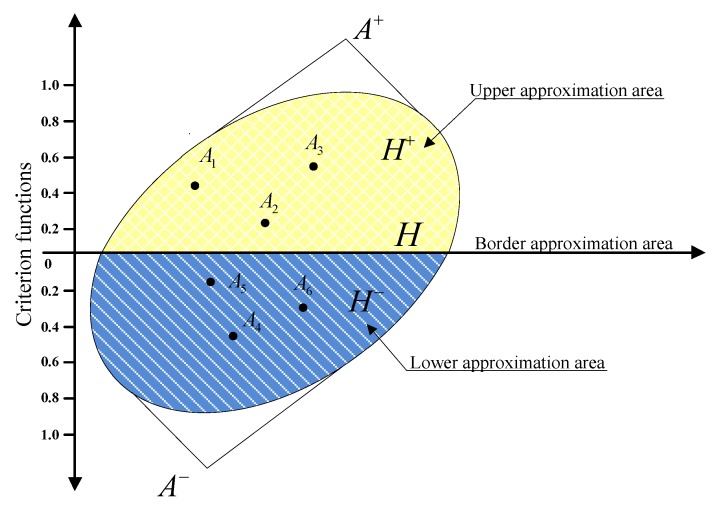
Presentation of the upper ($H^{+}$), lower ($H^{-}$ ) and border (H ) approximation areas.

If $d_{ij}=0$, the alternative $A_{i}$ could belong to the BAA matrix H. If $d_{ij}>0$, it may belong to the upper approximation area (UAA) $H^{+}$. If $d_{ij}<0$, it may belong to the lower approximation area (LAA) $H^{-}$. Therefore, the best alternative $A^{+}$ is contained within the UAA $H^{+}$, while the worst alternative $A^{-}$ is contained within the LAA $H^{-}$. The expression is shown in the following Figure 1 [32].

**Step 5.** Finally, by employing the overall value $d_{i}$, the alternatives can be ranked, where $d_{i}$ is defined by

(7) $$d_{i}=\sum_{j=1}^{n}d_{ij},\;i\in M.$$

The larger the value $d_{i}$, the better the alternative $A_{i}$.

## 3. Linguistic Distribution Power Average Operator

In order to handle the aggregating problem with abnormal data, motivated by the power average (PA) operator [35,36], we propose the power aggregating operator with linguistic distribution evaluations.

**Definition** **4.** *Suppose*$\Phi =\left\{\varphi_{1},\varphi_{2},\ldots ,\varphi_{q}\right\}$*is a set of linguistic distribution evaluations, where the linguistic distribution evaluation can be regarded as $\varphi_{k}=\left\{(s_{r},p_{r}^{k})|\sum_{r=0}^{\tau }p_{r}^{k}=1,r=0,1,\ldots ,\tau \right\}$. The linguistic distribution power average (LDPA) operator is defined as*

$$LDPA(\varphi_{1},\varphi_{2},\cdots ,\varphi_{q})=\overset{q}{\underset{k=1}{⊕}}\frac{\left(1+T(\varphi_{k})\right)}{\sum_{j=1}^{q}\left(1+T(\varphi_{k})\right)}\varphi_{k}$$

(8) $$=\left\{\left(s_{r},\sum_{k=1}^{q}p_{r}^{k}\frac{\left(1+T(\varphi_{k})\right)}{\sum_{k=1}^{q}\left(1+T(\varphi_{k})\right)}\right)|\sum_{r=0}^{\tau }\sum_{k=1}^{q}p_{r}^{k}\frac{\left(1+T(\varphi_{k})\right)}{\sum_{k=1}^{q}\left(1+T(\varphi_{k})\right)}=1,r=0,1,\cdots ,\tau \right\},$$

*where*$T(\varphi_{k})=\sum_{l=1,l\neq k}^{q}Sup(\varphi_{k},\varphi_{l})$*,*$Sup(\varphi_{k},\varphi_{l})=1-d(\varphi_{k},\varphi_{l})$*is the support degree of the elements*$\varphi_{k}$*and*$\varphi_{l}$, which satisfies the following properties:

(1) $Sup(\varphi_{k},\varphi_{l})\in \left[0,1\right]$;
(2) $Sup(\varphi_{k},\varphi_{l})=Sup(\varphi_{l},\varphi_{k})$;
(3) *If*$d(\varphi_{k},\varphi_{l})<d(\varphi_{k},\varphi_{i})$*, then*$Sup(\varphi_{k},\varphi_{l})>Sup(\varphi_{k},\varphi_{i})$.

*For convenience, let*$v_{k}=\frac{\left(1+T(\varphi_{k})\right)}{\sum_{k=1}^{q}\left(1+T(\varphi_{k})\right)}\left(k=1,2,\ldots ,q\right)$*. Therefore,*$LDPA(\varphi_{1},\varphi_{2},\cdots ,\varphi_{q})=\overset{q}{\underset{k=1}{⊕}}v_{k}\varphi_{k}=$$\left\{\left(s_{r},\sum_{k=1}^{q}p_{r}^{k}v_{k}\right)|\sum_{r=0}^{\tau }\sum_{k=1}^{q}p_{r}^{k}v_{k}=1,r=0,1,\cdots ,\tau \right\}$.

Based on the definition and theory, the properties of the LDPA operator are shown in the following.

**Property 4** **(Idempotence).** *Suppose*$\Phi =\left\{\varphi_{1},\varphi_{2},\ldots ,\varphi_{q}\right\}$*is a set of linguistic distribution evaluations, where*$\varphi_{k}=\{(s_{r},p_{r}^{k})|\sum_{r=0}^{\tau }p_{r}^{k}=1,r=0,1,\ldots ,\tau \}=\varphi =\{(s_{r},p_{r}^{})|\sum_{r=0}^{\tau }p_{r}^{}=1,r=0,1,\ldots ,\tau \}$*for*∀k=1,2,…,q*, then we have*$LDPA(\varphi_{1},\varphi_{2},\cdots ,\varphi_{q})=\varphi$.

The proof can be seen in Appendix A.

**Property 5** **(Commutativity).** *Let*$\Phi {}^{\prime }=\left\{\varphi_{1}{}^{\prime },\varphi_{2}{}^{\prime },\ldots ,\varphi_{q}{}^{\prime }\right\}$*be any permutation of*$\Phi =\left\{\varphi_{1},\varphi_{2},\ldots ,\varphi_{q}\right\}$*, where*$\varphi_{k}=\left\{(s_{r},p_{r}^{k})|\sum_{r=0}^{\tau }p_{r}^{k}=1,r=0,1,\ldots ,\tau \right\}$*, thus*$LDPA(\varphi_{1},\varphi_{2},\cdots ,\varphi_{q})=LDPA(\varphi_{1}{}^{\prime },\varphi_{2}{}^{\prime },\cdots ,\varphi_{q}{}^{\prime })$.

The proof can be seen in Appendix A.

**Property 6** **(Boundary).** *Let*$\Phi =\left\{\varphi_{1},\varphi_{2},\ldots ,\varphi_{q}\right\}$*be a set of linguistic distribution evaluations, where*$\varphi_{k}=\left\{(s_{r},p_{r}^{k})|\sum_{r=0}^{\tau }p_{r}^{k}=1,r=0,1,\ldots ,\tau \right\}$. $\varphi_{}^{+}=\left\{\left(s_{0},0),(s_{1},0),\cdots ,(s_{\tau },1\right)\right\}$*and*$\varphi_{}^{-}=\{(s_{0},1),(s_{1},0),\cdots ,(s_{\tau },0)\}$*, thus*$\varphi_{}^{-}\leq LDPA(\varphi_{1},\varphi_{2},\cdots ,\varphi_{q})\leq \varphi_{}^{+}$.

The proof can be seen in Appendix A.

In the following, the linguistic distribution weighted power average (LDWPA) operator is defined.

**Definition** **5.** *Suppose*$\Phi =\left\{\varphi_{1},\varphi_{2},\ldots ,\varphi_{q}\right\}$ is a set of linguistic distribution evaluations, where the linguistic distribution evaluation can be regarded as $\varphi_{k}=\left\{(s_{r},p_{r}^{k})|\sum_{r=0}^{\tau }p_{r}^{k}=1,r=0,1,\ldots ,\tau \right\}$*. The weights vector of the linguistic distribution evaluations is* $\lambda =(\lambda_{1},\lambda_{2},\cdots ,\lambda_{q})$. The linguistic distribution weighted power average (LDWPA) operator is defined as

$$LDWPA(\varphi_{1},\varphi_{2},\cdots ,\varphi_{q})=\overset{q}{\underset{k=1}{⊕}}\frac{\lambda_{k}^{}\left(1+T(\varphi_{k})\right)}{\sum_{k=1}^{q}\lambda_{k}^{}\left(1+T(\varphi_{k})\right)}\varphi_{k}$$

(9) $$=\left\{\left(s_{r},\sum_{k=1}^{q}p_{r}^{k}\frac{\lambda_{k}^{}\left(1+T(\varphi_{k})\right)}{\sum_{k=1}^{q}\lambda_{k}^{}\left(1+T(\varphi_{k})\right)}\right)|\sum_{r=0}^{\tau }\sum_{k=1}^{q}p_{r}^{k}\frac{\lambda_{k}^{}\left(1+T(\varphi_{k})\right)}{\sum_{k=1}^{q}\lambda_{k}^{}\left(1+T(\varphi_{k})\right)}=1,r=0,1,\cdots ,\tau \right\},$$

*where* $T(\varphi_{k})=\sum_{l=1,l\neq k}^{q}Sup(\varphi_{k},\varphi_{l})$*,* $Sup(\varphi_{k},\varphi_{l})=1-d(\varphi_{k},\varphi_{l})$ *is the support degree of the elements* $\varphi_{k}$ *and* $\varphi_{l}$, which satisfies the following properties:

(1) $Sup(\varphi_{k},\varphi_{l})\in \left[0,1\right]$;
(2) $Sup(\varphi_{k},\varphi_{l})=Sup(\varphi_{l},\varphi_{k})$;
(3) *If*$d(\varphi_{k},\varphi_{l})<d(\varphi_{k},\varphi_{i})$*, then*$Sup(\varphi_{k},\varphi_{l})\geq Sup(\varphi_{k},\varphi_{i})$.

*For convenience, let*$v_{k}=\frac{\left(1+T(\varphi_{k})\right)}{\sum_{k=1}^{q}\left(1+T(\varphi_{k})\right)}\left(k=1,2,\ldots ,q\right)$*. Therefore,*$LDPA(\varphi_{1},\varphi_{2},\cdots ,\varphi_{q})=\overset{q}{\underset{k=1}{⊕}}v_{k}\varphi_{k}$$=\left\{\left(s_{r},\sum_{k=1}^{q}p_{r}^{k}v_{k}\right)|\sum_{r=0}^{\tau }\sum_{k=1}^{q}p_{r}^{k}v_{k}=1,r=0,1,\cdots ,\tau \right\}$.

## 4. Emergency Decision Problem and Its Resolution Procedure

### 4.1. Formulation of the Emergency Decision Problem

Nowadays, multiple-group decisions, which is a complex process, plays an increasingly important role in emergency management. Considering an emergency decision problem, the most desirable emergency alternative needs to be selected. By research provided by experts from multiple groups, several acceptable emergency alternatives are determined, which constitute a set of emergency alternatives. However, different emergency alternatives have their own advantages and disadvantages for each criterion. Therefore, the emergency decision maker vacillates among several emergency alternatives. Besides, to support the selection of the decision maker, a great many experts from different groups need to evaluate alternatives concerning the criteria of the linguistic set. A brief description of the emergency decision problem is presented in the following.

As a matter of routine, the following notations are used in the problem throughout this paper. Let M={1,2,⋯,m}, N={1,2,⋯,n}, and Q={1,2,⋯,q} be three sets of subscripts. Considering an emergency alternatives selection problem, suppose $A=\{A_{1},A_{2},\cdots ,A_{m}\}$ with m≥2 being a finite set of emergency alternatives, where $A_{i}$ expresses the ith emergency alternative, i∈M. Additionally, $C=\{C_{1},C_{2},\cdots ,C_{n}\}$ with n≥2 being a finite set of emergency criteria, where $C_{j}$ expresses the jth evaluation criterion, j∈N. Generally, the criteria set C can be provided by the emergency decision maker. Here, we suppose that the weights vector of criteria is completely unknown. However, the prior order of emergency criteria is provided by the emergency decision maker. Without a loss of generality, we suppose $C_{1}>C_{2}>\cdots >C_{n}$. The criterion $C_{j}$ has a higher priority than criterion $C_{j{}^{\prime }}$ if $j<j{}^{\prime }$. In addition, suppose $S=\{s_{0},s_{1},\cdots ,s_{\tau }\}$ is a linguistic set employed to evaluate emergency alternatives. $D=\{D_{1},D_{2},\cdots ,D_{q}\}$ is a set of subgroups. Each subgroup consists of experts, represented as $D_{k}=\{d_{k}^{1},d_{k}^{2},\cdots ,d_{k}^{I_{k}}\}$, where $d_{k}^{t}$ is the *t*th expert from the *k*th subgroup, $t=1,2,\cdots ,I_{k}$, where $I_{k}$ is the number of experts for the *k*th subgroup. Then, the weight vector of subgroups is $\lambda =\{\lambda_{1},\lambda_{2},\cdots ,\lambda_{q}\}$, which is provided by the emergency decision maker.

Suppose $X^{\left(k)(t\right)}={\left[x_{ij}^{\left(k)(t\right)}\right]}_{m\times n}$ is the emergency decision matrix, k=1,2,⋯,q, and $t=1,2,\cdots ,I_{k}$, where $x_{ij}^{\left(k)(t\right)}$ is the linguistic term evaluation of emergency alternative $A_{i}$ for criterion $C_{j}$ provided by the *t*th expert from the *k*th subgroup. The emergency decision matrix will be constructed in this paper.

The problem addressed in this study is how to select the most desirable emergency alternative(s) from the finite emergency alternative set A based on the criteria evaluation with a criteria prior order, in which the evaluations of the emergency alternatives in the format of linguistics are provided by different experts from different subgroups.

### 4.2. Framework and Processing of the Problem

**Figure 2:**
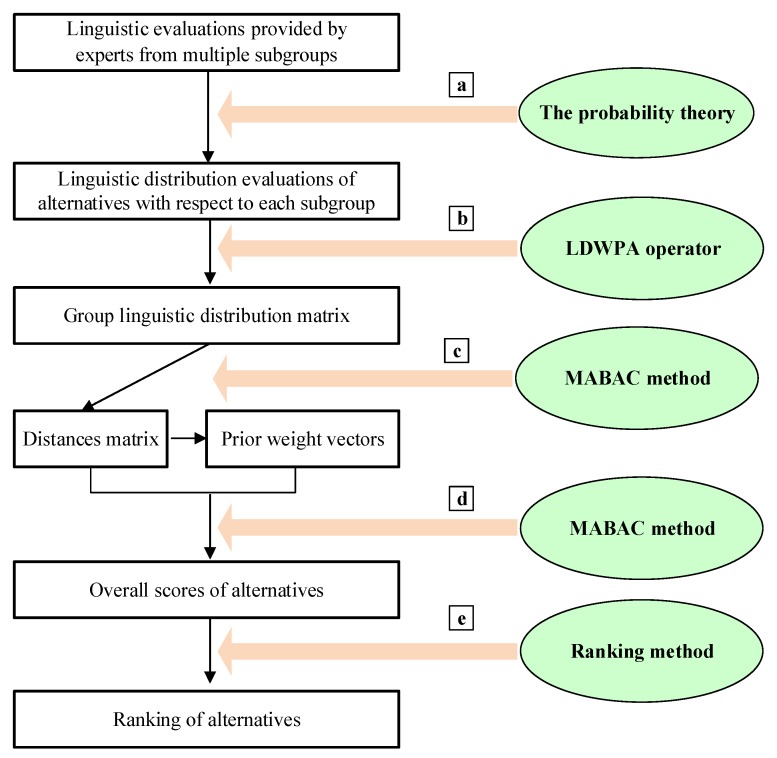
The resolution procedure for the emergency decision problem.

In order to solve the above emergency decision problem, a resolution process of ranking and selecting emergency alternatives is addressed, which is shown in Figure 2. It can be seen that the resolution process combines four steps. Considering the linguistic evaluations obtained from different experts from different subgroups, a ranking method for emergency alternatives is addressed. 

A brief expression of each step is shown below:

(a) According to the linguistic evaluations provided by a great many experts from multiple subgroups, evaluations of emergency alternatives in the format of a linguistic distribution can be constructed.
(b) According to the linguistic distribution evaluations of emergency alternatives associated with different subgroups, the group linguistic distribution evaluations are calculated. With respect to linguistic distribution evaluations provided by experts from different subgroups, due to the different benefits of different subgroups, the evaluations of emergency alternatives may be extreme. Therefore, a novel operator, known as the linguistic distribution weighted power (LDWPA) operator, is constructed to aggregate linguistic distribution evaluations provided by different subgroups.
(c) With respect to the group decision matrix, based on the LD-MABAC method, the distances matrix of alternatives for each criterion oriented to the lower approximation is calculated. Subsequently, considering the prior orders of the criteria, the method of determining the prior weights vector is developed.
(d) By aggregating the summing-up distance values for all criteria of the emergency alternatives, the comprehensive distance values of the alternatives can be obtained.
(e) Then, the ranking result of the alternatives is determined by comparing the comprehensive distance values.

## 5. A Novel Emergency Decision Method

On the basis of the resolution process shown in Figure 2, a specific implementation process of the novel method for ranking and selecting emergency alternatives will be represented in this section. In Section 5.1, a description of the part for constructing the linguistic distribution evaluations of the emergency alternatives associated with each subgroup follows. Then, the method for aggregating the subgroup evaluations of the emergency alternatives is established. On this basis, the group linguistic distribution evaluations of the emergency alternatives are computed. In Section 5.2, we present a novel LD-MABAC method. Then, the distances matrix of the emergency alternatives can be constructed. Next, based on the distribution linguistic evaluations and criteria prior order given by the emergency decision makers, the prior weights of the criteria are determined in Section 5.3. Finally, in Section 5.4, the method is presented to derive the ranking result of the emergency alternatives.

### 5.1. Calculation of the Group Evaluations for Emergency Alternatives

We suppose $S=\{s_{0},s_{1},\cdots ,s_{\tau }\}$ is a linguistic set provided by the emergency decision maker. To accurately describe the different evaluations provided by different experts, in this study, evaluations of emergency alternatives are represented as linguistic distribution variables. After obtaining the linguistic evaluations provided by experts form multiple subgroups, based on statistics theory, the evaluation associated with each subgroup expert is summarized.

Suppose $I_{k}$ is the number of linguistic evaluations for emergency alternative $A_{i}$ associated with criterion $C_{j}$ provided by the *k*th subgroup experts. Let $S=\{s_{0},s_{1},\cdots ,s_{\tau }\}$ be the $\varphi_{ij}^{\left(k\right)}=\{(s_{r},p_{ijr}^{\left(k\right)})|r=0,1,\cdots ,\tau \}$, in the format of a linguistic distribution, and be the evaluation value for emergency alternative $A_{i}$ associated with criterion $C_{j}$.

In the above linguistic distribution evaluations $\varphi_{ij}^{\left(k\right)}$, the probabilities $p_{ijr}^{\left(k\right)}$ are calculated by the following formula:

(10) $$p_{ijr}^{\left(k\right)}=\frac{1}{I_{k}}\sum_{t=1}^{I_{k}}\theta^{r}(x_{ij}^{\left(k)(t\right)}),\;i\in M,\;j\in N,\;k\in Q,\;r=0,1,\cdots ,\tau ,$$

where $\theta^{r}(x_{ij}^{\left(k)(t\right)})=\left\{ \begin{array}{l} 1,\;x_{ij}^{\left(k)(t\right)}=s_{r}\\ 0,\;x_{ij}^{\left(k)(t\right)}\neq s_{r} \end{array}\right.$, i∈M, j∈N, k∈Q, r=0,1,⋯,τ.

Based on the LDWPA operator, the overall group decision matrix $\Psi ={\left(\psi_{ij}^{}\right)}_{m\times n}$ of the emergency alternatives is calculated, in which $\psi_{ij}^{}=\{(s_{r},p_{ijr}^{})|r=0,1,\cdots ,\tau \}$ is represented in the following:

$$\;\psi_{ij}=\{(s_{r},p_{ijr}^{})|r=0,1,\cdots ,\tau \}=LDWPA(\varphi_{ij}^{1},\varphi_{ij}^{2},\cdots ,\varphi_{ij}^{q})=\overset{q}{\underset{k=1}{⊕}}\frac{\lambda_{k}^{}\left(1+T(\varphi_{ij}^{k})\right)}{\sum_{k=1}^{q}\lambda_{k}^{}\left(1+T(\varphi_{ij}^{k})\right)}\varphi_{ij}^{k}$$

(11) $$=\left\{\left(s_{r},\sum_{k=1}^{q}p_{ijr}^{\left(k\right)}\frac{\lambda_{k}^{}\left(1+T(\varphi_{ij}^{k})\right)}{\sum_{k=1}^{q}\lambda_{k}^{}\left(1+T(\varphi_{ij}^{k})\right)}\right)|\sum_{r=0}^{\tau }\sum_{k=1}^{q}p_{ijr}^{\left(k\right)}\frac{\lambda_{k}^{}\left(1+T(\varphi_{ij}^{k})\right)}{\sum_{k=1}^{q}\lambda_{k}^{}\left(1+T(\varphi_{ij}^{k})\right)}=1,r=0,1,\cdots ,\tau \right\},\;i\in M,j\in N,$$

where $T(\varphi_{ij}^{k})=\sum_{l=1,l\neq k}^{q}Sup((\varphi_{ij}^{k}),(\varphi_{ij}^{l}))$, $Sup(\varphi_{ij}^{k},\varphi_{ij}^{l})=1-d(\varphi_{ij}^{k},\varphi_{ij}^{l})$ is the support degree of the elements $\varphi_{ij}^{k}$ and $\varphi_{ij}^{l}$.

For convenience, let $v_{k}=\frac{\lambda_{k}^{}\left(1+T(\varphi_{ij}^{k})\right)}{\sum_{k=1}^{q}\lambda_{k}^{}\left(1+T(\varphi_{ij}^{k})\right)}\left(k=1,2,\ldots ,q\right)$. Therefore, $LDWPA(\varphi_{ij}^{1},\varphi_{ij}^{2},\cdots ,\varphi_{ij}^{q})$ $=\overset{q}{\underset{k=1}{⊕}}v_{k}\varphi_{ij}^{k}=\left\{\left(s_{r},\sum_{k=1}^{q}p_{ijr}^{\left(k\right)}v_{k}\right)|\sum_{r=0}^{\tau }\sum_{k=1}^{q}p_{ijr}^{\left(k\right)}v_{k}=1,r=0,1,\cdots ,\tau \right\}$.

### 5.2. Construction of the Distance Matrix

In this subsection, we propose a linguistic distribution MABAC (LD-MABAC) method, which is represented as follows. To begin with, we can determine the border approximation area (BAA) vector $H=(h_{1},h_{2},\cdots ,h_{n})$, where $h_{j}$ is calculated by

(12) $$h_{j}=LDA(\psi_{1j},\psi_{2j},\cdots ,\psi_{mj})=\{(s_{r},p_{jr}^{}),r=0,1,\cdots ,\tau \},\;j\in N,$$

where $p_{jr}^{}=\frac{1}{n}\sum_{i=1}^{n}p_{ijr}^{}$ satisfies $\sum_{r=0}^{\tau }\sum_{i=1}^{n}p_{ijr}^{}=1$.

Then, the distance matrix $D={\left(d_{ij}\right)}_{m\times n}$ can be constructed, where $d_{ij}$ is computed by

(13) $$d_{ij}=\left\{ \begin{array}{ll} d(\psi_{ij},h_{j}), & if\;E(\psi_{ij})>E(h_{j})\\ 0, & if\;E(\psi_{ij})=E(h_{j})\\ -d(\psi_{ij},h_{j}), & if\;E(\psi_{ij})<E(h_{j}) \end{array}\right.,\;i\in M,\;j\in N,$$

where $d(\psi_{ij},h_{j})$ denotes the distance between the group evaluation $\psi_{ij}$ and the value $h_{j}$ in H.

Now, the alternative $A_{i}$ could belong to the BAA matrix H if $d_{ij}=0$, the upper approximation area (UAA) $H^{+}$ if $d_{ij}>0$, or the lower approximation area (LAA) $H^{-}$ if $d_{ij}<0$. Obviously, the best alternative $A^{+}$ is contained within the UAA $H^{+}$, whereas the worst alternative $A^{-}$ is contained within the LAA $H^{-}$ (Figure 1).

### 5.3. Determination of the Prior Criteria Weights

Due to the different importance of the evaluation criteria, the prior order of the criteria can be provided by the emergency decision maker. Without a loss of generality, we suppose $C_{1}>C_{2}>\cdots >C_{n}$. In order to aggregate the distance values $d_{ij}$ on all criteria into a comprehensive distance value $d_{i}$, the prior weight vector Ω should be determined. In the following, we determine the prior weight $\omega_{j}^{i}$ of the criterion $C_{j}$, based on the distance value $d_{ij}$ of alternative $A_{i}$.

Without a loss of generality, using the alternative $A_{i}$, for example, we suppose that the weight of the highest prior criterion $C_{1}$ is $\sigma_{1}^{i}=1$. Then, we define the other relative prior weight $\sigma_{j}^{i}$ of criterion $C_{j}$:

(14) $$\sigma_{j}^{i}=\sigma_{j-1}^{i}f_{i,j-1}=\prod_{g=1}^{j-1}f_{ig},\;i\in M,\;j\in N,\;j\neq 1.$$

In the above formula, a linear transformation function is used to transform the distance value $d_{ij}$ into $f_{ij}$, i.e.,

(15) $$f_{ij}=\frac{1}{2}(d_{ij}+1),\;i\in M,\;j\in N.$$

Furthermore, the relative prior weight $\sigma_{j}^{i}$ of prior criterion $C_{j}$ can be normalized into prior weight $\omega_{j}^{i}$:

(16) $$\omega_{j}^{i}=\frac{\sigma_{j}^{i}}{\sum_{j=1}^{n}\sigma_{j}^{i}},\;i\in M,\;j\in N.$$

According to Equations (14)–(16), we have some properties for prior weights, which are summarized in the following.

**Property** **7.** *For*∀i∈M*,*∀j∈N*,*$f_{ij}$*increases monotonically as the distance value*$d_{ij}$*. Specifically, the transformed distance value satisfies*$0<f_{ij}\leq 1$*and*$f_{ij}{}^{{}^{\prime }}>0$.

The proof can be seen in Appendix A.

**Property** **8.** The weight of the high prior criterion is not less than the weight of the lower prior criterion. Specifically, for ∀i∈M*,* ∀j,l∈N*, if* $C_{j}>C_{l}$*,* j<l*, then we have* $\omega_{j}^{i}\geq \omega_{l}^{i}$.

The proof can be seen in Appendix A.

### 5.4. Obtainment of the Ranking Result for the Emergency Alternatives

When the prior weights of evaluation criteria are determined, based on the values of the emergency alternatives for each criterion, the comprehensive value $d_{i}$ of alternative $A_{i}$ can be calculated as

(17) $$d_{i}=\sum_{j=1}^{n}d_{ij},\;i\in M.$$

According to comprehensive value $d_{i}$, the ranking of emergency alternatives can be obtained. A larger comprehensive value $d_{i}$ indicates that $A_{i}$ is a better emergency alternative.

Summarizing the above analysis process, the decision procedures of the proposed method for handling the selection problem with linguistic distribution evaluations can be concluded as follows:

**Step 1.** Based on Equation (10), the evaluation in the format of a linguistic distribution matrix $\Phi^{\left(k\right)}={\left(\varphi_{ij}^{\left(k\right)}\right)}_{m\times n}$ associated with the *k*th subgroup is constructed.

**Step 2.** According to Equation (11), the group decision matrix $\Psi ={\left(\psi_{ij}^{}\right)}_{m\times n}$ is constructed.

**Step 3.** According to Equations (12) and (13), the distance matrix of emergency alternatives $D={\left(d_{ij}\right)}_{m\times n}$ can be calculated.

**Step 4.** The prior weight vector $\Omega^{i}={\left(\omega_{1}^{i},\omega_{2}^{i},\cdots ,\omega_{n}^{i}\right)}^{T}$ associated with emergency alternative $A_{i}$ can be determined according to Equations (14)–(16).

**Step 5.** The comprehensive value $d_{i}$ of the emergency alternative $A_{i}$ can be calculated according to Equation (17).

**Step 6.** The alternatives ranking result can be determined.

## 6. A Case Study

### 6.1. A Description of the Case

In order to explain the practicality of the novel proposed method, a case of evaluating emergency alternatives is shown.

In the summer flood season, precipitation is frequent. Affected by the cloud system outside the typhoon, a city has been experiencing continuous rainfall in recent days, and local rainstorms occur from time to time. Because there are many low-lying areas in the urban area of the city, the possibility of urban waterlogging disasters has risen sharply. In order to do a good job in urban flood prevention and rescue work during the flood season and maintain the safety of citizens’ lives and property, as emergency decision makers, local government groups assume the urban geological conditions and drainage facilities based on the weather conditions predicted by meteorological experts and the continuous rainfall conditions. Referring to the situations of many historical events in the past, the city’s emergency decision makers extracted the following four emergency response plans from the emergency plan database:

$A_{1}$: Radio and television stations are rolling out warning messages to remind residents to properly handle outdoor items. Outworkers stay away from electrified facilities and dangerous buildings and avoid low-lying roads. Relevant groups clean the road in time.

$A_{2}$: Some low-lying areas are closed to traffic. Remind citizens to go out less. Take protective measures for outdoor work, such as high-altitude work, and reinforce outdoor devices.

$A_{3}$: Some low-lying areas are closed to traffic. Citizens are told not to go outside and avoid outdoor personnel nearby. Check the safety condition of factories and other facilities in dangerous industries, such as industry and mining, and cut off the power supply in dangerous areas.

$A_{4}$: Evacuate people from low-lying areas. Some units stop production and shutdown. Some schools are closed.

Considering the following five criteria to evaluate the above four emergency alternatives, the decision makers employ multiple subgroup experts to evaluate the emergency alternatives associated with multiple criteria with linguistic terms by adopting the linguistic terms set $S=\{s_{0}=terrible,\;s_{1}=low,s_{2}=ordinary,\;s_{3}=high,s_{4}=perfect\}$. Then, the set of emergency decision criteria is {$C_{1}$: Emergency rescuing effect; $C_{2}$: Emergency responding time; $C_{3}$: Emergency preparing capacity; $C_{4}$: Direct economic loss; $C_{5}$: Emergency input cost}. The emergency decision maker employs experts from three departments, which are regarded as three subgroups: the environment department, ministry of health, and ministry of materials and equipment. The weight vector of the four subgroups is provided by the emergency decision maker: $\lambda ={\left(1/3,1/3,1/3\right)}^{T}$. Each department involves ten experts to evaluate the emergency alternatives with linguistic variables by the linguistic terms set S.

### 6.2. Methodology and Results

First, we obtained the linguistic evaluations from multiple subgroups provided by experts. Due to the original linguistic evaluations being obtained, including uncertainty and being massive, having been provided by a great many experts from multiple subgroups, we converted the original linguistic evaluations into linguistic distribution evaluations. Therefore, the total numbers of linguistic evaluations were counted, based on the probability theory, and we could construct linguistic distribution evaluations. Then, based on Equation (10), the evaluations for emergency alternative *A_i_* concerning criterion $C_{j}$ associated with subgroup *D_k_*, which is regarded by linguistic distribution variable $\varphi_{ij}^{\left(k\right)}=\{(s_{r},p_{ij}^{r(k)})|r=0,1,\cdots ,\tau \}$, could be constructed.

Based on the weighted linguistic distribution power operator, i.e., Equation (11), the group linguistic distribution evaluations $\psi_{ij}^{}$ of alternative $A_{i}$ concerning criterion $C_{j}$ could be obtained. Given the limited space, we detail the evaluations provided by three subgroups for emergency alternative $A_{1}$ for criterion $C_{1}$. Specifically, the evaluations from three subgroups for emergency alternative $A_{1}$ for criterion $C_{1}$ are $\varphi_{11}^{\left(1\right)}=\{(s_{0},0.1),(s_{1},0.3),(s_{2},0.3),(s_{3},0.2),(s_{4},0.1)\}$, $\varphi_{11}^{\left(2\right)}=\{(s_{0},0)$, $\left(s_{1},0.1),(s_{2},0.3),(s_{3},0.5),(s_{4},0.1)\right\}$, $\varphi_{11}^{\left(3\right)}=\{(s_{0},0.1),(s_{1},0.2),(s_{2},0.3),(s_{3},0.4),(s_{4},0)\}$. Therefore, based on Equation (11), the group linguistic distribution evaluations $\psi_{11}^{}$ of alternative $A_{1}$ concerning criterion $C_{1}$ could be aggregated as: $\psi_{11}^{}=\{(s_{0},0.0675),(s_{1},0.2011),(s_{2},0.3),(s_{3},0.3652),(s_{4},0.0662)\}$. In this way, the distribution linguistic decision matrix with respect to the four emergency alternatives on the five criteria could be formed, which is shown in the following Table 1, Table 2, Table 3, Table 4 and Table 5.

On this basis, by using Equations (12) and (13), the distance value $d_{ij}$ of alternative $A_{i}$ for criterion $C_{j}$ with respect to border approximation area vector $H={\left(h_{1},h_{2},h_{3},h_{4},h_{5}\right)}^{T}$, the distance matrix can be shown as follows:

$$D={\left[d_{ij}\right]}_{4\times 5}=\left(\begin{array}{lllll} 0 & 0 & 0 & -0.2206 & -0.2287\\ 0 & 0 & 0.0999 & -0.2101 & -0.1545\\ 0 & 0.0880 & 0 & -0.1072 & -0.1458\\ -0.0913 & 0.0442 & 0 & -0.3234 & -0.2374 \end{array}\right)$$

According to the prior order of criteria, as well as Equations (14)–(16), the prior weights of criteria associated with emergency alternatives could be calculated as $\Omega^{1}={\left(0.5198,0.2599,0.1300,0.0650,0.0253\right)}^{T}$,$\Omega^{2}={\left(0.5150,0.2575,0.1287,0.0708,0.0280\right)}^{T}$, $\Omega^{3}={\left(0.5079,0.2540,0.1382,0.0691,0.0308\right)}^{T}$ and $\Omega^{4}={\left(0.5405,0.2455,0.1282,0.0641,0.0217\right)}^{T}$. After aggregating the distance values of emergency alternatives associated with all criteria, by using Equation (17), the overall distance values are shown as

$$d_{1}=-0.0201,\;d_{2}=-0.0063,\;d_{3}=0.0105,\;d_{4}=-0.0644$$

For the value $d_{i}$, the larger the value $d_{i}$, the better the alternative $A_{i}$. Therefore, the ranking result of the four alternatives could be derived as follows:

$$A_{3}>A_{2}>A_{1}>A_{4}$$

Therefore, the most desirable emergency alternative was $A_{3}$.

### 6.3. Sensitivity Analysis

In this subsection, a sensitivity analysis of the proposed method, which was combined with the LDWPA operator and LD-MABAC, is presented, to verify the stability of the proposed method. The ranking results of the emergency alternatives depend, to some extent, on the weight of the experts or criteria. Sometimes, the final selections change when there is a very slight change in the weight of the experts or criteria. Therefore, a sensitivity analysis was performed to assess how changes in the weights assigned to the subgroups would change the ranking of the emergency alternatives. We considered the following scenarios associated with the different weight vectors of the subgroups: $\lambda_{1}={\left(1/3,1/3,1/3\right)}^{T}$, $\lambda_{2}={\left(0.4,0.3,0.3\right)}^{T}$, $\lambda_{3}={\left(0.3,0.4,0.3\right)}^{T}$, $\lambda_{4}={\left(0.3,0.3,0.4\right)}^{T}$, $\lambda_{5}={\left(1/2,1/2,0\right)}^{T}$, $\lambda_{6}={\left(1/2,0,1/2\right)}^{T}$ and $\lambda_{7}={\left(0,1/2,1/2\right)}^{T}$. Corresponding to different weights assigned to each subgroup, the ranking results are shown in Table 6.

From the ranking results in Table 6, we can see that the different weights of the subgroups slightly lead to different ranking results of emergency alternatives; that is, the decision method is slightly sensitive to these weights. However, in most scenarios, the best emergency alternatives are the same. Additionally, the trend in ranking is roughly the same in Table 6. A large change in the ranking results is only obtained in the few scenarios with extreme weights for the subgroups. Therefore, the method presented in this paper has a good stability, and we can use different weights to confirm rankings so as to select the best alternative. However, there is not enough data available on which to base a conclusion related to the reliability of the results for the sensitivity of these methods to changes in the weight of the subgroups. In order to further illustrate the advantages of the proposed method, we will compile a comparative analysis in the following Subsection.

### 6.4. Comparative Analysis

In this subsection, in order to show the feasibility of the proposed method, we add a new example from the Liu et al. [37] for demonstration.

**Example from** (Liu et al. [37]): Consider an evaluation problem with the teacher appointment system reformation in a university. Some teachers from seven schools $G_{1},\;G_{2},\;G_{3},\;G_{4},\;G_{5},\;G_{6},\;G_{7}$ can login into the system and provide his/her personal preference for three reform alternatives. Each teacher selects one score from the pre-established score set $S=\{s_{0},\;s_{1},s_{2},s_{3},s_{4}\}$. The weight vector of the subgroups is $\lambda ={\left(0.14,0.16,0.18,0.14,0.11,0.13,0.14\right)}^{T}$. Then, the evaluations of the alternatives are formulated as the distribution information presented in Table 7.

In the following, we compare the proposed LDWPA operator and operational laws of linguistic distribution in this study with the previously designed ones by Liu et al. [37] and the linguistic distribution weighted average (LDWA) operator provided by Zhang et al. [27]. The problem formulation and comparison process are shown in Table 8.

As shown in Table 8, the trends of the ranking results by using the mentioned methods are basically in line with each other, while the first ranking result is different from the others. With respect to the first method provided by Zhang et al. [27], the aggregation operator was slightly rough, involving additive compensation. The results from the method proposed by Liu et al. [37] and in this study are the same, which can illustrate the practicability of the proposed method in this study.

Furthermore, taking the example in this paper again, we respectively used the LDWPA operator, the LDWA operator (Zhang et al. [27]), and the method proposed by Liu et al. [37] combined with the LD-MABAC method in this study to illustrate the feasibility of the methods. Additionally, we used the proposed LDWPA operator in this study combined with the LD-VIKOR (Liang et al. [31]) method for solving the example. To ensure comparability of these methods, we used the same weights vector of criteria $\Omega ={\left(0.45,0.25,0.15,0.1,0.05\right)}^{T}$. Therefore, the comparative results are shown in Table 9.

As shown in Table 9, the trends of the ranking results by using the mentioned methods are basically in line with each other, while the first and the third ranking results are different from the others. On the one hand, in the first three ranking results, similar to those in Table 8, a similar problem arises in the method provided by Zhang et al. [27]. The ranking result from Zhang et al. [27] and LD-MABAC is different from that of the other two methods, which further explains the rationality of the proposed LDWPA operator in this study. The main reason for this is that the LDWPA operator can aggregate some extreme information situations effectively. On the other hand, in the last two ranking results, we can see that the LDWPA operator combined with LD-MABAC in this study can obtain clear and unambiguous ranking results. In spite of the fact that the general trend of the last ranking result is the same as that obtained by the proposed method in this study, due to the flaw of the LD-VIKOR method, only the set of compromised solution alternatives can be derived.

Summarizing the above analysis, the method of combining the LDWPA operator and LD-MABAC proposed in this study has a good stability and effectiveness for solving multiple-group emergency decision problems. In addition, compared with existing methods, the proposed method in this study can also solve the problem in which the weights of the criteria are unknown while just having the prior order.

## 7. Conclusions

The decision problem for emergency alternatives selection with linguistic evaluation provided by multiple-group experts has profound theoretical value and practical implications. In order to select emergency alternatives, a practical decision analysis method needs to be investigated. In this study, aimed at the problem of emergency alternatives selection, a decision analysis method was proposed. In the novel method, by aggregating evaluations for emergency alternatives provided by experts from multiple groups, quantitative information can be prepared for the decision-making process. This provides a valid tool for solving multiple-group emergency decision problems with linguistic evaluation information. Based on the LD-MABAC method, the distance matrix can be calculated. Subsequently, the prior weight is determined so as to obtain the ranking result.

Compared with existing research, this study has the following obvious contributions. On the one hand, from an application perspective, the emergency alternatives selection problem has been discussed to derive a novel decision model to support selection. The emergency decision-making problem involving multiple-group experts was investigated, avoiding evaluation unification. On the other hand, two operators, known as LDPA and LDWPA operators, for handling linguistic distribution evaluation information, were developed. Then, linguistic evaluations provided by experts associated with different groups were aggregated based on novel operators. The novel operators can handle extreme evaluations. The linguistic distribution evaluation distance of emergency alternatives was developed. The LD-MABAC method for handling decision-making problems with distribution linguistics was proposed. This proposed method can avoid the information loss or distortion that occurs in most existing methods.

In future research, in order to enrich the research results, it is worth noting that criteria aspirations provided by experts from different groups can be considered. Furthermore, considering that the evaluation criteria are not completely known, a study on handling missing information needs to be conducted. Additionally, different utility values of the evaluations obtained from different experts can be incorporated into future investigations.

## Figures and Tables

**Table 1:** The linguistic distribution evaluations on criterion $C_{1}$.

Alternatives	Evaluations in Format of Linguistic Distribution Variables				
	$s_{0}$	$s_{1}$	$s_{2}$	$s_{3}$	$s_{4}$
$A_{1}$	0.0675	0.2011	0.3000	0.3652	0.0662
$A_{2}$	0.0000	0.1005	0.3333	0.3671	0.1991
$A_{3}$	0.0331	0.0331	0.3013	0.4656	0.1669
$A_{4}$	0.000$s_{0}$0	0.0673	0.2014	0.3993	0.3320

**Table 2:** The linguistic distribution evaluations on criterion $C_{2}$.

Alternatives	Evaluations in Format of Linguistic Distribution Variables				
	$s_{0}$	$s_{1}$	$s_{2}$	$s_{3}$	$s_{4}$
$A_{1}$	0.0330	0.0670	0.2332	0.3662	0.3006
$A_{2}$	0.0674	0.4009	0.3339	0.1313	0.0665
$A_{3}$	0.1662	0.2351	0.3991	0.1667	0.0329
$A_{4}$	0.166$s_{0}$4	0.2309	0.2324	0.3367	0.0336

**Table 3:** The linguistic distribution evaluations on criterion $C_{3}$.

Alternatives	Evaluations in Format of Linguistic Distribution Variables				
	$s_{0}$	$s_{1}$	$s_{2}$	$s_{3}$	$s_{4}$
$A_{1}$	0.0328	0.0328	0.2674	0.2333	0.4337
$A_{2}$	0.0332	0.0666	0.2670	0.3666	0.2666
$A_{3}$	0.0664	0.2338	0.2330	0.3325	0.1343
$A_{4}$	0.3338	0.1336	0.2991	0.2000	0.0334

**Table 4:** The linguistic distribution evaluations on criterion $C_{4}$.

Alternatives	Evaluations in Format of Linguistic Distribution Variables				
	$s_{0}$	$s_{1}$	$s_{2}$	$s_{3}$	$s_{4}$
$A_{1}$	0.3723	0.4968	0.0986	0.0323	0
$A_{2}$	0.5671	0.2329	0	0.2000	0
$A_{3}$	0.0665	0.1991	0.3337	0.3670	0.0337
$A_{4}$	0.0334	0.1000	0.2332	0.1332	0.5002

**Table 5:** The linguistic distribution evaluations on criterion $C_{5}$.

Alternatives	Evaluations in Format of Linguistic Distribution Variables				
	$s_{0}$	$s_{1}$	$s_{2}$	$s_{3}$	$s_{4}$
$A_{1}$	0	0.0679	0.1330	0.4661	0.3330
$A_{2}$	0.0330	0.0674	0.2663	0.3657	0.2676
$A_{3}$	0.1339	0.3996	0.2996	0.1004	0.0665
$A_{4}$	0.3669	0.2331	0.2662	0.1007	0.0331

**Table 6:** The ranking results associated with the different weights of the subgroups.

Scenarios	Ranking Results	Scenarios	Ranking Results
$\lambda_{1}={\left(1/3,1/3,1/3\right)}^{T}$	$A_{3}>A_{2}>A_{1}>A_{4}$	$\lambda_{5}={\left(1/2,1/2,0\right)}^{T}$	$A_{3}>A_{4}>A_{1}>A_{2}$
$\lambda_{2}={\left(0.4,0.3,0.3\right)}^{T}$	$A_{3}>A_{1}>A_{4}>A_{2}$	$\lambda_{6}={\left(1/2,0,1/2\right)}^{T}$	$A_{4}>A_{3}>A_{2}>A_{1}$
$\lambda_{3}={\left(0.3,0.4,0.3\right)}^{T}$	$A_{3}>A_{2}>A_{1}>A_{4}$	$\lambda_{7}={\left(0,1/2,1/2\right)}^{T}$	$A_{2}>A_{3}>A_{4}>A_{1}$
$\lambda_{4}={\left(0.3,0.3,0.4\right)}^{T}$	$A_{3}>A_{2}>A_{1}>A_{4}$		

**Table 7:** The distribution evaluations of the alternatives.

Alternatives	Rating Scales	Subgroups						
		$G_{1}$	$G_{2}$	$G_{3}$	$G_{4}$	$G_{5}$	$G_{6}$	$G_{7}$
$A_{1}$	$s_{0}$	0.018	0.096	0.007	0.104	0.337	0.346	0.174
	$s_{1}$	0.045	0.16	0.028	0.224	0.292	0.308	0.33
	$s_{2}$	0.116	0.24	0.197	0.259	0.18	0.192	0.33
	$s_{3}$	0.357	0.288	0.282	0.241	0.135	0.096	0.105
	$s_{4}$	0.464	0.216	0.486	0.172	0.056	0.058	0.061
$A_{2}$	$s_{0}$	0.509	0.176	0.127	0.086	0.079	0.125	0.061
	$s_{1}$	0.366	0.256	0.155	0.155	0.135	0.144	0.139
	$s_{2}$	0.062	0.24	0.247	0.31	0.202	0.192	0.191
	$s_{3}$	0.036	0.2	0.239	0.233	0.247	0.25	0.279
	$s_{4}$	0.027	0.128	0.232	0.216	0.337	0.289	0.33
$A_{3}$	$s_{0}$	0.036	0.112	0.373	0.397	0.067	0.058	0.07
	$s_{1}$	0.098	0.256	0.232	0.224	0.146	0.154	0.096
	$s_{2}$	0.223	0.264	0.113	0.181	0.191	0.211	0.191
	$s_{3}$	0.429	0.272	0.127	0.129	0.236	0.231	0.226
	$s_{4}$	0.214	0.096	0.155	0.069	0.34	0.346	0.417

**Table 8:** Comparison of the results obtained using the three methods.

Methods	Ranking of Alternatives
The LDWA operator presented by Zhang et al. [27]	$A_{1}>A_{3}>A_{2}$
The method presented by Liu et al. [37]	$A_{3}>A_{2}>A_{1}$
The proposed LDWPA operator in this paper	$A_{3}>A_{2}>A_{1}$

**Table 9:** Comparison of the results obtained using the four combined methods.

Methods	Ranking of Alternatives
The method presented by Liu et al. [37] and classical MABAC	$A_{3}>A_{2}>A_{1}>A_{4}$
The LDWA operator presented by Zhang et al. [27] and LD-MABAC	$A_{1}>A_{3}>A_{2}>A_{4}$
The proposed method of combining the LDWPA operator and LD-MABAC	$A_{3}>A_{2}>A_{1}>A_{4}$
The method of combining the LDWPA operator and LD-VIKOR (Liang et al. [31])	$\{A_{1},A_{2},A_{3}\}>A_{4}$

## Appendix A

**Proof of** **Property 4.** Since $\varphi_{k}=\{(s_{r},p_{r}^{k})|\sum_{r=0}^{\tau }p_{r}^{k}=1,r=0,1,\ldots ,\tau \}=\varphi =\{(s_{r},p_{r}^{})|\sum_{r=0}^{\tau }p_{r}^{}=1,r=0,1,\ldots ,\tau \}$ for ∀k=1,2,…,q, then we have

$$\begin{array}{ll} LDPA(\varphi_{1},\varphi_{2},\cdots ,\varphi_{q}) & =\left\{\left(s_{r},\sum_{k=1}^{q}p_{r}^{k}\frac{\left(1+T(\varphi_{k})\right)}{\sum_{k=1}^{q}\left(1+T(\varphi_{k})\right)}\right)|\sum_{r=0}^{\tau }\sum_{k=1}^{q}p_{r}^{k}\frac{\left(1+T(\varphi_{k})\right)}{\sum_{k=1}^{q}\left(1+T(\varphi_{k})\right)}=1,r=0,1,\cdots ,\tau \right\}\\ & =\left\{\left(s_{r},\sum_{k=1}^{q}p_{r}\frac{\left(1+T(\varphi_{k})\right)}{q\left(1+T(\varphi_{k})\right)}\right)|\sum_{r=0}^{\tau }p_{r}\frac{\left(1+T(\varphi_{k})\right)}{q\left(1+T(\varphi_{k})\right)}=1,r=0,1,\cdots ,\tau \right\}\\ & =\left\{\left(s_{r},p_{r}\right)|\sum_{r=0}^{\tau }p_{r}=1,r=0,1,\cdots ,\tau \right\}=\varphi \end{array}$$

 □

**Proof of** **Property 5.** Since $\Phi {}^{\prime }=\left\{\varphi_{1}{}^{\prime },\varphi_{2}{}^{\prime },\ldots ,\varphi_{q}{}^{\prime }\right\}$ can be any permutation of $\Phi =\left\{\varphi_{1},\varphi_{2},\ldots ,\varphi_{q}\right\}$, $\sum_{k=1}^{q}\left(1+T(\varphi_{k})\right)=\sum_{k=1}^{q}\left(1+T(\varphi_{k}{}^{\prime })\right)$ and $\sum_{k=1}^{q}\left(1+T(\varphi_{k})\right)p_{r}^{k}=\sum_{k=1}^{q}\left(1+T(\varphi_{k}{}^{\prime })\right)p_{r}^{k}{}^{\prime }$; that is, $\sum_{k=1}^{q}\frac{\left(1+T(\varphi_{k})\right)}{\sum_{k=1}^{q}\left(1+T(\varphi_{k})\right)}p_{r}^{k}=\sum_{k=1}^{q}\frac{\left(1+T(\varphi_{k}{}^{\prime })\right)}{\sum_{k=1}^{q}\left(1+T(\varphi_{k}{}^{\prime })\right)}p_{r}^{k}{}^{\prime }$. Therefore, we have

$$\begin{array}{ll} LDPA(\varphi_{1},\varphi_{2},\cdots ,\varphi_{q}) & =\left\{\left(s_{r},\sum_{k=1}^{q}p_{r}^{k}\frac{\left(1+T(\varphi_{k})\right)}{\sum_{k=1}^{q}\left(1+T(\varphi_{k})\right)}\right)|\sum_{r=0}^{\tau }\sum_{k=1}^{q}p_{r}^{k}\frac{\left(1+T(\varphi_{k})\right)}{\sum_{k=1}^{q}\left(1+T(\varphi_{k})\right)}=1,r=0,1,\cdots ,\tau \right\}\\ & =\left\{\left(s_{r},\sum_{k=1}^{q}p_{r}^{k{}^{\prime }}\frac{\left(1+T(\varphi_{k}{}^{\prime })\right)}{\sum_{k=1}^{q}\left(1+T(\varphi_{k}{}^{\prime })\right)}\right)|\sum_{r=0}^{\tau }\sum_{k=1}^{q}p_{r}^{k{}^{\prime }}\frac{\left(1+T(\varphi_{k}{}^{\prime })\right)}{\sum_{k=1}^{q}\left(1+T(\varphi_{k}{}^{\prime })\right)}=1,r=0,1,\cdots ,\tau \right\}\\ & =LDPA(\varphi_{1}{}^{\prime },\varphi_{2}{}^{\prime },\cdots ,\varphi_{q}{}^{\prime }) \end{array}$$

 □

**Proof of** **Property 6.** According to Definition 2 of the linguistic distribution, we can obtain that $E(\varphi_{}^{-})=s_{0}$, $E(\varphi_{}^{-})=s_{\tau }$ and

$$0\leq \Delta \left(E\left(LDPA(\varphi_{1},\varphi_{2},\cdots ,\varphi_{q})\right)\right)=\sum_{r=0}^{\tau }r\left(\sum_{k=1}^{q}\frac{\left(1+T(\varphi_{k})\right)}{\sum_{k=1}^{q}\left(1+T(\varphi_{k})\right)}p_{r}^{k}\right)\leq \sum_{r=0}^{\tau }\tau \cdot \left(\sum_{k=1}^{q}\frac{\left(1+T(\varphi_{k})\right)}{\sum_{k=1}^{q}\left(1+T(\varphi_{k})\right)}p_{r}^{k}\right)=\tau$$

Here, Δ(⋅) is the subscript of the linguistic term. Then we have $s_{0}\leq E(LDPA(\varphi_{1},\varphi_{2},\cdots ,\varphi_{q}))\leq s_{\tau }$. Therefore, we can obtain $\varphi_{}^{-}\leq LDPA(\varphi_{1},\varphi_{2},\cdots ,\varphi_{q})\leq \varphi_{}^{+}$.□

**Proof** **of Property 7.** Since ∀i∈M and ∀j∈N, we have $f_{ij}=\frac{1}{2}(d_{ij}+1)$. Therefore, we have $0<f_{ij}\leq 1$, $f_{ij}{}^{{}^{\prime }}=\frac{1}{2}>0$, where $f_{ij}$ increases monotonically as the distance value $d_{ij}$. □

**Proof** **of Property 8.** Since $C_{j}>C_{l}$ for j<l, then we have $\sigma_{l}^{i}=\prod_{q=1}^{l-1}f_{iq}=\sigma_{j}^{i}\prod_{q=j}^{l-1}f_{iq}$ based on Equation (14), and $\prod_{q=j}^{l-1}f_{iq}\leq 1$ based on Equation (15). Therefore, $\sigma_{j}^{i}\geq \sigma_{l}^{i}$. Based on Equation (16), we have $\omega_{j}^{i}\geq \omega_{l}^{i}$. The weight of the high prior criterion is not less than the weight of the lower prior criterion. □
